# α-Glucosidase-driven metabolism as a potential therapeutic vulnerability in *Candida albicans*

**DOI:** 10.3389/fmicb.2026.1812644

**Published:** 2026-04-13

**Authors:** Joyati Mitra, Anusree Sajeevan, Swathi Sujith, Sasvin Paramasivam, Karthi Shanmugam, Adline Princy Solomon

**Affiliations:** 1 Quorum Sensing Laboratory, Centre for Research in Infectious Diseases, School of Chemical and Biotechnology, SASTRA Deemed University, Thanjavur, Tamil Nadu, India; 2 Department of Bioinformatics, School of Chemical and Biotechnology, SASTRA Deemed University, Thanjavur, Tamil Nadu, India

**Keywords:** acarbose, antifungal resistance, anti-virulence, *C. albicans*, α-glucosidase

## Abstract

*Candida albicans* remains a leading etiological agent of both mucosal and invasive fungal infections. The increasing prevalence of antifungal resistance and limited therapeutic options pose a significant clinical challenge and highlight the need for novel drug targets. The metabolic plasticity of *C. albicans* is closely linked to its pathogenicity, as the utilization of diverse carbon sources influences cell wall biogenesis, virulence factor development, and immune evasion. The most significant one is the metabolic enzyme α-glucosidase, which connects carbohydrate metabolism to the processing of N-glycans and the maturation of mannoproteins, contributing to cell wall integrity, adhesion, biofilm formation, and host-pathogen interactions. This article critically evaluates α-glucosidase as a potential metabolic and virulence-associated vulnerability in *C. albicans*. We combine knowledge of glycoside hydrolase family classification, catalytic mechanisms, the functional roles of α-glucosidases across different organisms, and a specific comparative study of fungal versus human enzymes. The phylogenetic and structural superimposition studies reveal a major evolutionary and three-dimensional divergence between fungal GH13 α-glucosidases and human GH31 homologs, despite the preservation of important catalytic sites. These differences create exploitable structural and physicochemical distinctions in the substrate-binding environment, providing a basis for the rational design of selective antifungal inhibitors with minimal off-target activity against human enzymes. Based on our previous research, we reported that the repurposing potential of α-glucosidase inhibitors, particularly acarbose, to demonstrate anti-virulence and antibiofilm activity against *C. albicans* at concentrations of 90–200 nM, and that these effects were synergistic when combined with existing antifungal agents. This review highlights that *in silico* modeling, docking studies, and targeted delivery strategies are beneficial tools that drive the development of α-glucosidase-based antifungal therapies. Collectively, this review underscores α-glucosidase-driven metabolism as a potential therapeutic vulnerability in *C. albicans*, providing a foundation for the rational drug design of metabolism-targeted antifungal strategies to overcome resistance and improve clinical outcomes.

## 1 Introduction: targeting metabolic and cell wall pathways in *Candida albicans*

**FIGURE 1:**
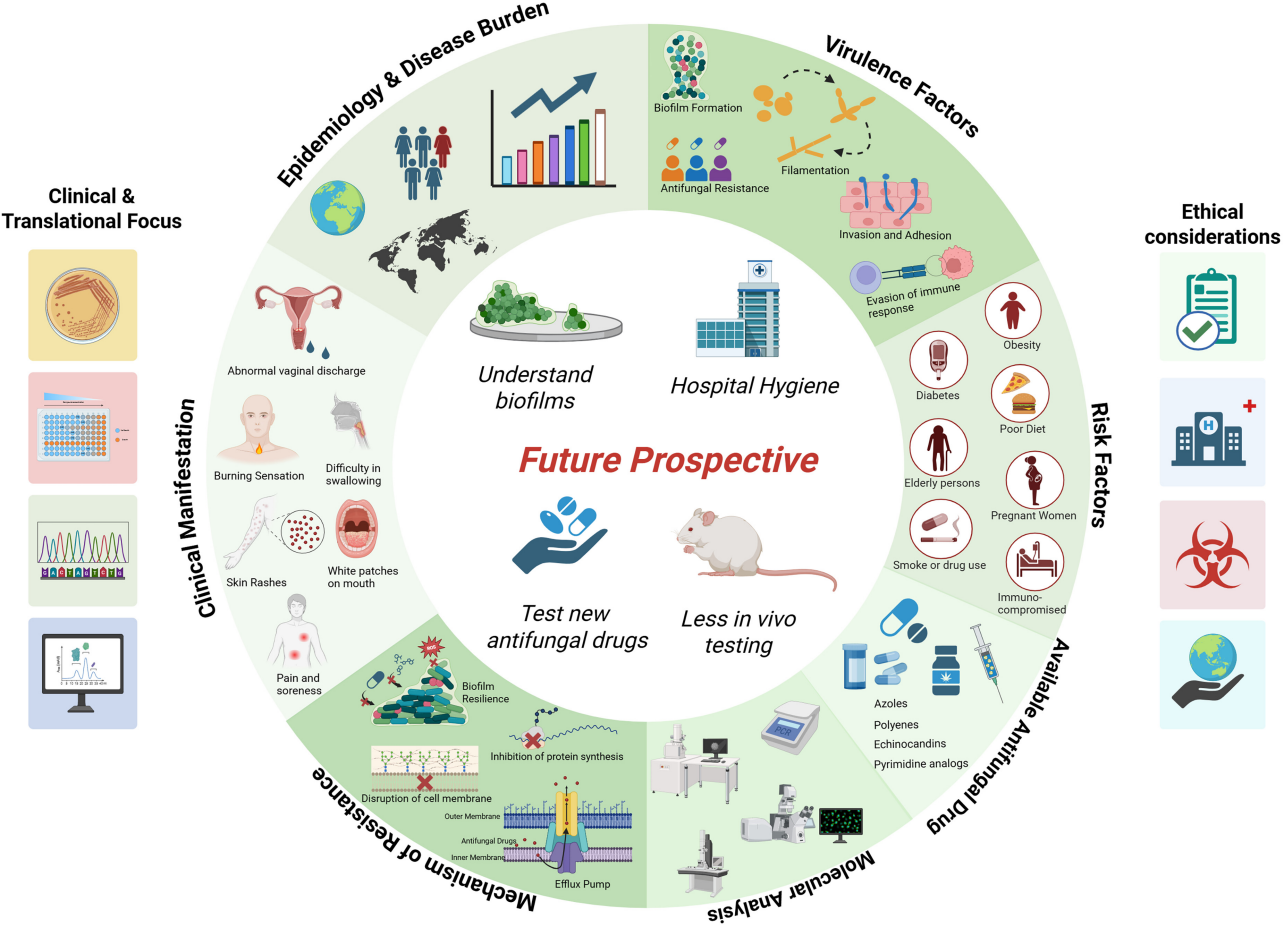
Overview of candidiasis pathogenesis, clinical impact, therapeutic challenges, and future perspectives. Created with BioRender.com.

*Candida albicans* is the leading cause of mucosal and systemic fungal infections, accounting for approximately 70% of candidiasis cases worldwide (Talapko et al., 2021). Although it is a commensal in healthy individuals, *C. albicans* can cause superficial infections such as oral thrush and vulvovaginal candidiasis. In immunocompromised hosts, however, it frequently progresses to an invasive and life-threatening disease (Pierce and Lopez-Ribot, 2013; Pfaller and Diekema, 2007). *C. albicans* is a major cause of hospital-acquired infections, contributing to approximately 15% of sepsis cases and nearly 40% of bloodstream infections reported in clinical studies (Basmaciyan et al., 2019). Collectively, *Candida* species rank among the third or fourth-leading causes of bloodstream infections among hospitalized patients (Edmond et al., 1999). In the United States alone, the annual incidence of systemic candidiasis is estimated at ∼20 cases per 100,000 individuals, with rates increasing up to 50-fold in high-risk hospitalized populations (Pierce and Lopez-Ribot, 2013). Among women of reproductive age, vulvovaginal candidiasis and its recurrent form remain highly prevalent. Persistent or recurrent infections involving the skin and mucosal surfaces are classified as chronic mucocutaneous candidiasis (CMC), predominantly affecting individuals with primary or acquired immunodeficiency disorders (David et al., 2024; Kühbacher et al., 2017). Immunosuppression, the use of broad-spectrum antibiotics, breach of the epithelial barrier, cytotoxic treatment, low birth weight, aging, AIDS, diabetes, and substance abuse are predetermining factors of candidiasis (Pierce and Lopez-Ribot, 2013). The close association of candidiasis with systemic illnesses has extensive clinical implications (Vila et al., 2020). *C. albicans* has become an important topic of worldwide health concern due to its growing clinical load, and it was identified as an urgent priority fungus by the World Health Organization (WHO) in 2022 (Fisher and Denning, 2023). The development of antifungal drugs has been marked by a very slow pace, owing to the limited number of fungal-specific molecular targets and the rapid emergence of multidrug-resistant fungal strains, which have caused greater morbidity and mortality (Kilbas et al., 2025). The existing clinical practice for the management of invasive fungi is based on four main antifungal groups, namely, polyenes, azoles, echinocandins and pyrimidine analogs (Pierce and Lopez-Ribot, 2013). Amphotericin B is one of the oldest antifungal agents, a polyene with a broad spectrum of action; however, its clinical use is constrained by severe toxicity (Roemer and Krysan, 2014). Azoles block fungal membrane sterol biosynthesis by inhibiting cytochrome P450 lanosterol 14-α-demethylase (Erg11), but are mostly fungistatic against *Candida* species (Lee et al., 2023). Increasing resistance to echinocandins has been reported across multiple *Candida* species, progressively compromising the clinical efficacy of this relatively newer class of antifungal agents (Perlin, 2015). The emergence of intrinsically drug-resistant non-*albicans Candida* species, compounded by the development of adaptive resistance in *C. albicans*, underscores the urgent need to develop novel therapeutic approaches that lack conventional drug targets (Arastehfar et al., 2020). *C. albicans* exhibits remarkable pathogenic adaptability beyond biofilm formation, employing multiple virulence strategies including pH adaptation, phenotypic switching, host cell adhesion and yeast-to-hypha morphological transitions (Martins et al., 2014). Collectively, these mechanisms synergistically contribute to its invasive potential and persistence within host tissues. These processes are closely associated with metabolic flexibility that enables the fungus to live in a variety of host niches. Carbohydrate utilization is one of the most important metabolic pathways in fungi, determining their survival, virulence, and immune evasion. There is dynamic regulation of carbon metabolism in *C. albicans* in response to nutrient availability within the host environment, controlled at the transcriptional level by factors such as Tye7 and Gal4, which regulate glycolysis, fermentation, and hexose catabolism. This metabolic adaptability not only supports growth but also directly influences cell wall composition and architecture, thereby linking metabolism to virulence. Attacking fungal metabolism is a promising alternative to the conventional approach to antifungal responses. Contrary to the membrane- or cell-wall-directed drugs, metabolic interventions may concomitantly interfere with energy production, biosynthesis, and virulence pathways, and this may decrease the selective pressure of resistance. The main carbon source for *C. albicans* is carbohydrates, which are metabolized in central metabolism as glucose-6-phosphate or fructose-6-phosphate after enzymatic processing. The enzymes involved in carbohydrate processing are, hence, metabolic bottlenecks that can be selectively targeted using antifungal interventions. The carbohydrates form the major source of carbon used to generate energy and biosynthesize building blocks in fungi, with the majority of the sugars being broken down to glucose-6-phosphate or fructose-6-phosphate and then fed into glycolysis (Askew et al., 2009). The presence of carbohydrates like glucose and galactose has a significant role in host-pathogen interactions as it brings about metabolic reprograming that favors immune response, commensalism, or pathogenic growth (Pellon et al., 2022). In this connection, it can be stated that the biology of infection and the role of metabolic adaptation and the nutritional environment became widely recognized in the last decade (Traven and Naderer, 2019). On the contrary, the metabolism of carbohydrates in the human host is dependent on digestive enzymes that break down carbohydrates and help them to be absorbed. The brush border epithelial cells of the intestine express alpha-glucosidase, a major enzyme that degrades disaccharides and oligosaccharides into monosaccharides that can be absorbed (Fatmawati et al., 2011). α-Glucosidase has long been recognized as a critical target for controlling postprandial hyperglycemia in diabetes, leading to the development of α-glucosidase inhibitors (AGIs) for the management of type II diabetes mellitus (T2DM) (Pan et al., 2024; Qi et al., 2016). AGIs, such as acarbose and voglibose, delay carbohydrate digestion and absorption, thereby reducing glycemic spikes after a meal (Yonekura et al., 2024). The first clinically approved AGI, acarbose, is an extract of *Actinomyces utaensis*, which acts as a competitive inhibitor of α-glucosidase (Wehmeier and Piepersberg, 2003). In addition to its role in host metabolism, α-glucosidase has been shown to play a significant role in *C. albicans* cell wall biogenesis and virulence. *C. albicans* contains glycosylphosphatidylinositol (GPI)-linked mannoproteins cross-linked to 8 glucans on the outer cell wall layer. N-linked mannans are made of an α-16-mannan backbone with α-1,2-oligomannoside chains terminated by β-1,2-linked mannose residues (Siafakas et al., 2007). The antigenicity of the protein, adhesion, and immune recognition depend on mannoproteins processed by α-glucosidase (David et al., 2024). Interference with α-glucosidase-mediated N-glycan processing disturbs the cell wall integrity, morphology, and virulence in *C. albicans* (Mora-Montes et al., 2007). Recent advances in understanding *C. albicans* virulence have highlighted multiple metabolic and cell wall-associated pathways as promising targets for antifungal and anti-virulence drug development (David and Solomon, 2023). Interestingly, it has been experimentally demonstrated that acarbose acts as an effective inhibitor of *C. albicans* pathogenicity by suppressing fungal growth rather than exerting direct fungicidal activity, as confirmed by minimum inhibitory concentration (MIC) and minimum biofilm inhibitory concentration (MBIC) analyses showing significant inhibition of growth and biofilm formation (David et al., 2024). Conversely, there are constraints associated with the use of acarbose, whether delivered systemically or orally, for the treatment of oral candidiasis, especially given its pharmacokinetic profile and potential complications in patients with comorbid conditions. Comparative structural and functional characterization of fungal and human α-glucosidases enables the identification of fungus-specific features that can be exploited to rationally design selective antifungal inhibitors. Structure-guided optimization of existing α-glucosidase inhibitors, including acarbose, informed by differences between fungal and human enzymes, may improve antifungal potency while reducing off-target effects on host enzymes. Therefore, this review provides a comparative structural and functional assessment of fungal and human α-glucosidases to identify exploitable distinctions that may facilitate the design of selective therapeutic strategies against *C. albicans*–associated candidiasis (Figure 1).

## 2 Glycoside hydrolase families and α-glucosidase diversity

### 2.1 Overview of GH31 and GH13 families

Glycoside hydrolase family 31 (GH31) is a highly versatile group of enzymes with α-glucosidases, α-xylosidases, isomaltosyltransferases, and maltase/glucoamylases having high-substrate specificity and biological activity (Kawano et al., 2019; Arumapperuma et al., 2023). The Enzyme Commission (EC) classification defines α-glucosidases of this family as those that catalyze the exohydrolysis of 1,4-α-glycosidic linkages in maltooligosaccharides, releasing α-D-glucose. GH31 α-glucosidases commonly possess dual catalytic capabilities, mediating both hydrolysis and transglucosylation. The latter reaction facilitates the synthesis of oligosaccharides with α-1,2, α-1,3, and α-1,6 glycosidic linkages, highlighting the functional diversity and biosynthetic potential of this enzyme family (Kawano et al., 2019). GH31 α-glucosidases are involved in numerous crucial biological functions, such as dietary carbohydrate digestion, lysosomal catabolism of glycoconjugates, and post-translational processing of glycoproteins (Kawano et al., 2019). In mammals, carbohydrate digestion involves the α-glucosidases in the brush border of intestinal enterocytes, which hydrolyze the α-glycosidic bonds of disaccharides and oligosaccharides. These enzymes have reiterated GH31 catalytic domains, which are highly important for substrate processing (Jongkees and Withers, 2013; Lombard et al., 2014). GH31 enzymes are retaining α-glucosidases that catalyze an acid/base reaction via a covalent glycosyl-enzyme complex, thereby maintaining the anomeric structure of the liberated glucose (Lovering et al., 2005). Notably, one member of this family is human lysosomal α-glucosidase, the deficiency of which causes Pompe disease (glycogen storage disease type II), highlighting the physiological irreplaceability of GH31 enzymes (Lovering et al., 2005). The GH13 family, whose members exhibit a broad range of catalytically diverse glycoside hydrolysis, is another significant branch in the glycoside hydrolase superfamily, and its catalytic repertoire and sequence diversity are further expanded by its functional diversity, as evidenced by the GH31 family. The α-amylase family or GH13 is the largest glycoside hydrolase family and has a broad specificity toward α-glycosidic bonds, including α-glucosidases, α-glucan lyases, α-xylanases and sulfoquinovosidases (Stam et al., 2006). A multiscale, systematic examination of 1,691 GH13 sequences, using clustering, sequence-based analysis, and phylogenetic reconstruction, yielded a robust subfamily-level classification. This comparison indicated that about 80% of the sequence was predictably classified into 35 smaller subfamilies, most of which were monofunctional, suggesting a strong association between sequence preservation, substrate specificity, and the outcome of catalysis (Stam et al., 2006). In GH13, several closely related subfamilies are the only active alpha-amylases with high internal sequence homology, but they diverge significantly, unlike other GH13 groups, indicating evolutionary pressure to maintain essential structural and catalytic elements (Plaza-Vinuesa et al., 2019). The family is further indicated to have diverse evolutionary and physiological significance by the presence of GH13 α-glucosidases in fungi, bacteria, and other eukaryotes (Table 1).

**TABLE 1:** GH-31 and GH-13 family α-glucosidase isoforms, domain structure, localization, and function.

Isoform/enzyme gene		PDB	Uniprot ID	Experimental method	Organism	Molecular weight (kDa)	Domain structure	Localization	Substrate specificity	Physiological function	Associated disease	References
GH-31 Family	Lysosomal acid α-glucosidase (GAA)	5NN8	P10253	X-RAY DIFFRACTION (2.45 Å)	*Homo sapiens*	∼95–110	•Contains a GH31 catalytic domain along with multiple glycosylated domains. •Undergoesproteolytic processing to form the mature lysosomal enzyme.	Lysosome	•Cleaves terminal α-1,4 linkage. •Can hydrolyze α-1,6 linkages in glycogen and related glucans.	Cleavage of the α-1,4- and α-1,6-glycosidic bonds of glycogen to glucose.	Pompe disease (GSD II)	(Lim et al., 2014)
	Maltase-glucoamylase (MGAM)	2QMJ	O43451	X-RAY DIFFRACTION (1.9 Å)	*Homo sapiens*	∼160	•Terminal catalytic domain. •Small cytosolic domain •Transmembrane domain anchoring into brush border membrane	Intestinal brush border	Maltose, maltotriose	Hydrolysis of short oligosaccharides	Carbohydrate maldigestion	(Sim et al., 2008)
	Sucrase–Isomaltase (SI)	3LPO	P14410	X-RAY DIFFRACTION (3.2 Å)	*Homo sapiens*	∼245	•Two catalytic domains: Sucrase (C-terminal) Isomaltase (N terminal) •Both belong to glycoside hydrolase family 31 (GH31)	Apical membrane of small intestinal brush border	•Sucrase: hydrolyzes sucrose •Maltose Isomaltase: hydrolyzes α-1,6 linkages (isomaltose, branched oligosaccharides)	Final digestion of dietary starch and sucrose into glucose + fructose	Congenital Sucrase-Isomaltase Deficiency (CSID)	(Gericke et al., 2017)
	Neutral α-glucosidase	8D43	Q14697	ELECTRON MICROSCOPY (2.88 Å)	*Homo sapiens*	167.31 kDa	–	Epididymis	Hydrolysis of α-glucosidic bonds in neutral pH substrates	•Marker of epididymal function •Reflects sperm maturation and epididymal patency	Reduced activity associated with epididymal dysfunction	(Eertmans et al., 2014)
GH-13 Family	α-Glucosidase GSJ	2ZE0	Q33390	X-RAY DIFFRACTION (2.00 Å)	*Geobacillus sp. HTA-462*	65.3 kDa	•Typical GH13 fold (α/β domain architecture characteristic of family)—active-site residues D, E, D conserved	Bacterial cytosolic/periplasmic	α-1,4-linked glucosides (exoglycosidic cleavage)—releases glucose from non-reducing ends	•Final step in starch/glucan degradation •Carbohydrate metabolism in bacterium	–	(Shirai et al., 2008)
	Isomaltase	3A47	P53051	X-RAY DIFFRACTION (1.59 Å)	*Saccharomyces cerevisiae*	68.72 kDa	•Belong to the α-glucosidase (AGase) family •Share conserved “region II” though a Thr → Val substitution in region II distinguishes IMA enzymes.	Yeast intracellular/ extracellular	The Thr→Val substitution in region II is linked to a shift in specificity from α-1,4 to α-1,6.	•Metabolism of α-1,6-linked disaccharides •Supports yeast carbohydrate utilization and metabolic flexibility.	–	(Teste et al., 2010)
	α-glucosidase-maltose	3WY4	H3K096	X-RAY DIFFRACTION (2.50Å)	*Halomonas sp. H11*	123.09 kDa	Typical GH13 architecture: (β/α)*8*-barrel catalytic domain + auxiliary domains (domains B/B’, C) forming the active-site pocket.	–	•α-1,4-Glucosidic linkages •Hydrolysis of glucosides •Structure defined with bound glucosyl intermediate	•Hydrolysis of terminal α-1,4-linked glucose residues •Provides insight into substrate binding and catalytic mechanism.	–	(Shen et al., 2015)
	α-glucosidase-maltotriose	7DCG	–	X-RAY DIFFRACTION (1.53 Å)	*Weissella cibaria*	70.19 kDa	•Typical GH13 architecture: a (β/α)_8_-barrel catalytic domain plus auxiliary domain(s) •Active site formed by barrel + associated loops/auxiliary domain(s)	Bacterial enzyme—likely cytosolic or secreted by *Weissella*	•Short-chain maltooligosaccharides (α-1,4 linkages) •Lacks activity on cyclic oligosaccharides or large polysaccharides	•Hydrolysis of small α-1,4-glucosidic linkages •Trans-glucosylation activity observed with maltose as acceptor	–	(Wangpaiboon et al., 2021)
	Glucosidase I (ER α-glucosidase I)	–	Q02751	–	*Candida albicans*	Fragment approx 50–60 kDa in that assay.	•Belongs to the glycosyl hydrolase family •Key catalytic domain in the C-terminal portion •N-terminal region likely for targeting/folding/ER localization.	Endoplasmic reticulum lumen	Removes the outermost α1,2-linked glucose from the Glc*3*Man*9*GlcNAc*2* core oligosaccharide on nascent glycoproteins.	•Critical for proper N-glycosylation •Affects cell wall composition •Influences folding of secreted proteins Maintains cell wall integrity	–	(Frade-Pérez et al., 2010)

## 3 Catalytic mechanism of α-glucosidases

**FIGURE 2:**
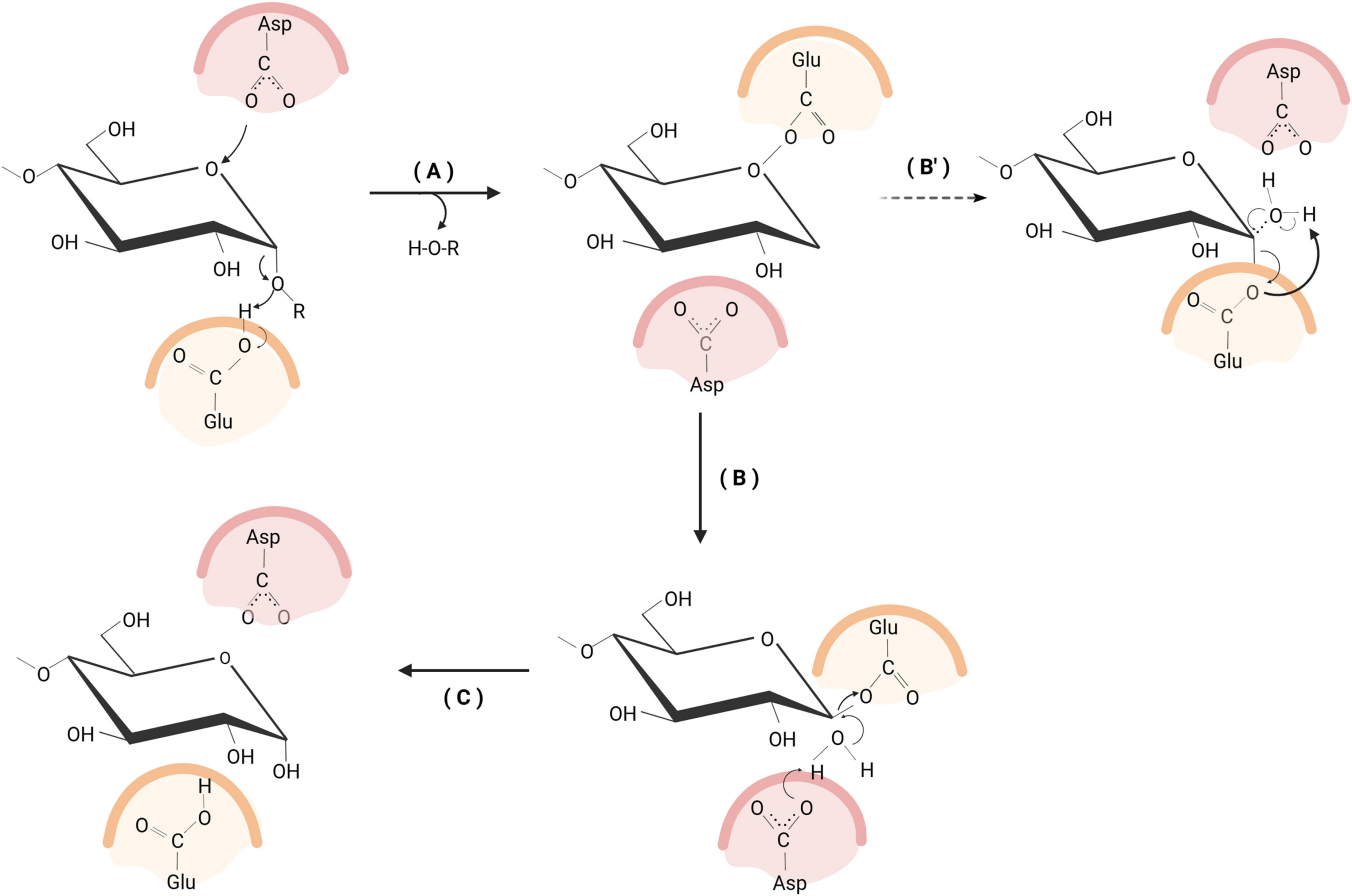
Retaining double-displacement mechanism of α-glucosidase. **(A)** Substrate binding and protonation of the glycosidic oxygen by glutamate. **(B)** Aspartate-mediated nucleophilic attack forming a covalent β-glucosyl-enzyme intermediate. **(B′)** Water activation by glutamate for nucleophilic attack. **(C)** Hydrolysis of the intermediate, releasing glucose with retention of α-configuration. Created with BioRender.com.

α-Glucosidases operate via a classical retaining double-displacement mechanism involving two conserved acidic residues, an aspartate (nucleophile) and a glutamate (acid/base catalyst). When the substrate is bound, the glutamate residue protonates the glycosidic oxygen and cleaves the α-1,4- or α-1,6 glycosidic bond. At the same time, the aspartate residue attacks the anomeric carbon (C1), forming a covalent β-glucosyl-enzyme intermediate and releasing the aglycone. During the deglycosylation step, the catalytic glutamate residue functions as a general base, activating a water molecule for nucleophilic attack on the anomeric carbon of the covalent glycosyl-enzyme intermediate. This reaction hydrolyzes the Asp–glucosyl bond, regenerates the catalytic residues, and releases free glucose while retaining the α-anomeric configuration. Throughout the catalytic cycle, the Asp and Glu side chains undergo coordinated protonation and deprotonation, ensuring efficient catalysis and stereochemical fidelity (Figure 2).

## 4 Pathogenic mechanism of *Candida albicans* via α-glucosidase and glycoprotein maturation

**FIGURE 3:**
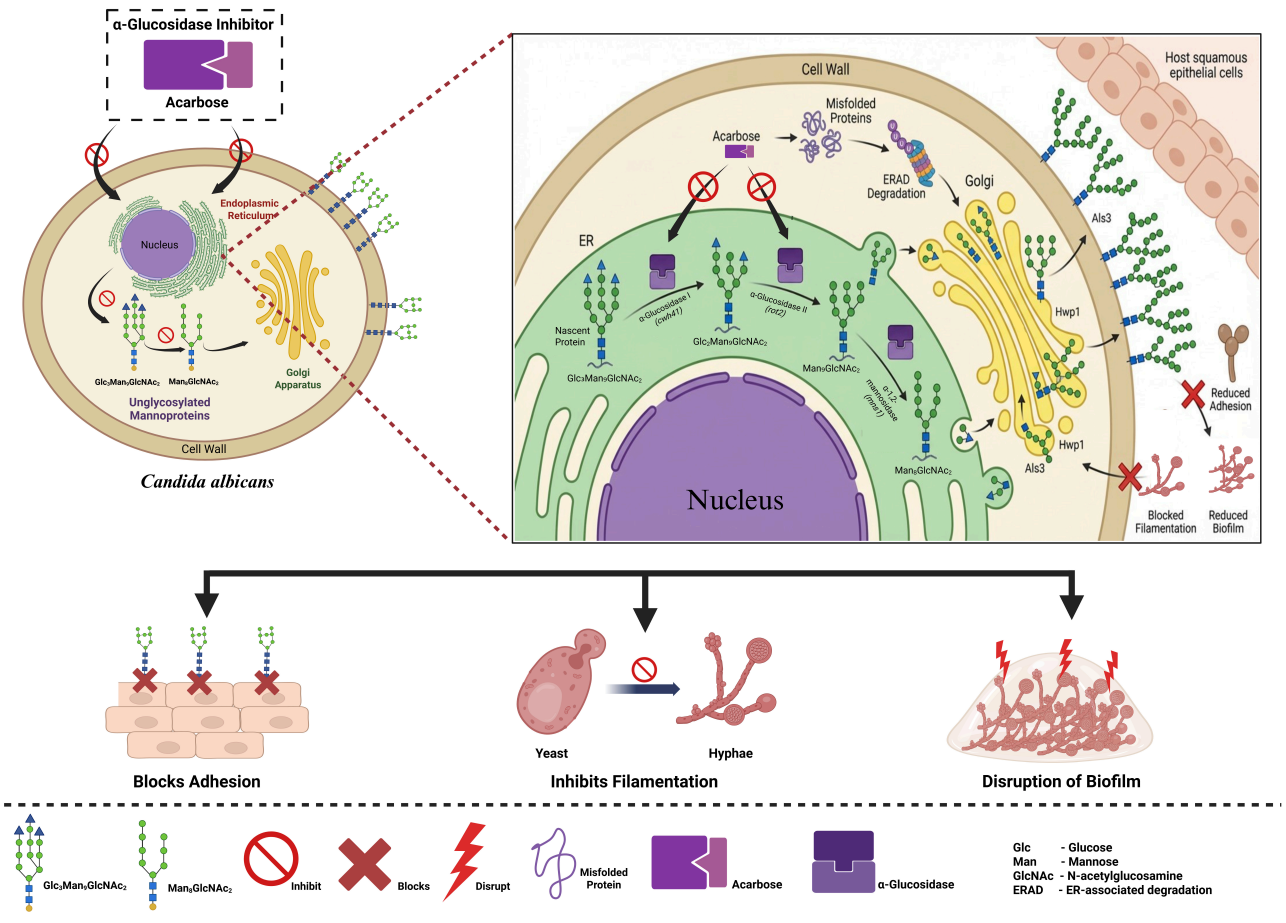
Pathogenic mechanism of *Candida albicans* via α-glucosidase and glycoprotein maturation. Created with BioRender.com.

For *C. albicans*, glycoprotein maturation in the secretory pathway is a significant process that supports cell wall integrity and contributes to virulence. As shown in Figure 3, the process begins in the endoplasmic reticulum (ER), in which newly synthesized nascent polypeptides enter the lumen and bind to a preassembled oligosaccharide precursor molecule that contains a Glc_3_Man_9_GlcNAc_2_ structure. The first step in glycoprotein maturation in *C. albicans* is catalyzed by the *cwh1* gene, which encodes α-glucosidase I. This enzyme removes the outermost glucose molecule from the precursor, producing a product containing the Glc_2_Man_9_GlcNAc_2_ structure. The next step in glycoprotein maturation in *C. albicans* is carried out by *rot2*, which encodes α-Glucosidase II. This enzyme sequentially removes the remaining two glucose residues from the precursor molecule, producing a product containing a Man_9_GlcNAc_2_ structure and then a final product containing a Man_8_GlcNAc_2_ structure. These sequential steps in glycoprotein maturation in *C. albicans* are important for ER quality-control mechanisms that ensure proper protein folding and subsequent transport from the ER to the golgi apparatus. In the golgi apparatus, enzymes like the α-1,2-mannosidase of the *mns1* convert the oligosaccharide chains, leading to the formation of mature mannoproteins. These mature mannoproteins then reach the cell membrane and integrate into the cell wall. These mannoproteins, such as Als3 and Hwp1, function as adhesins, helping the fungus attach to host epithelial cells and transition from yeast to hyphal form as it forms a biofilm. These are the major processes that increase the pathogenicity of the fungus. However, with the introduction of the α-glucosidase inhibitor acarbose, the process is disrupted. The inhibitor disrupts α-glucosidase enzymes in the ER, including those encoded by the *cwh41* and *rot2* genes. This disrupts the sequential process of glucose group removal from the oligosaccharide precursor. This means that glycoproteins such as Glc_3_Man_9_GlcNAc_2_, Glc_2_Man_9_GlcNAc_2_, and Man_9_GlcNAc_2_ will not be completely converted to the mature Man_8_GlcNAc_2_. These glycoproteins will not fold into their correct shapes and will be recognized by the ER. They will then be retained in the ER and degraded through the ER-associated degradation. As a result, the necessary mannoproteins cannot reach the cell surface. This affects the virulence of the organism. The inability of adhesins like Als3 and Hwp1 to mature correctly results in the disruption of the cell wall and significantly affects the ability of the organism to adhere to the tissues. This disruption of the cell wall affects the yeast-to-hyphal transition and biofilm formation thereby compromising the virulence of the organism.

## 5 Physiological roles of α-glucosidases in different organisms

### 5.1 Plants

α-glucosidases are pivotal in plant seed germination, mobilizing storage starch. They hydrolyze oligosaccharides produced by α-amylase, β-amylase and debranching enzymes to free glucose (Stanley et al., 2011). In addition to soluble substrates, plant α-glucosidases can hydrolyze the insoluble starch granules, which work in synergy with α-amylases to assist in breaking down the starch in seeds (Frandsen and Svensson, 1998; Sun and Henson, 1990). Genomic analyses have revealed species-specific differences in α-glucosidase gene organization, with barley possessing a single gene regulated by gibberellic acid, whereas spinach contains one gene that gives rise to multiple α-glucosidase isoforms, likely through post-translational modifications (Sugimoto et al., 1997).

### 5.2 Insects

In insects, carbohydrate metabolism requires α-glucosidase, which has been reported to play a role in arboviral infections, specifically Dengue virus serotype 2 (DENV-2). The expression of α-glucosidase in the midguts of the mosquitoes is increased during DENV-2 infection, which helps the virus to survive, replicate, and be transmitted (Tchankouo-Nguetcheu et al., 2010). The replication of DENV-2 is inhibited by pharmacological α-glucosidase, which demonstrates the possibility of this approach to regulating the spread of the virus in insect vectors (Bakhshi et al., 2018).

### 5.3 Bacteria

In bacteria, maltose and maltooligosaccharides are broken down by α-glucosidases that cleave off terminal α-1,4-glucosidic bonds, releasing glucose to be utilized in glycolysis and energy generation (Okuyama et al., 2016). The thermophilic bacterium Thermus thermophilus synthesizes a thermostable α-glucosidase enzyme with an optimal pH of 6.2 and a temperature of 85°C, which is applicable in industrial starch processing. Very close analogs of these enzymes are found in *Sulfolobus solfataricus* and *Thermococcus* species (Tomasik and Horton, 2012).

### 5.4 Mammals

The release of α-glucosidases by the intestinal epithelium plays a crucial role in the conversion of polysaccharides into monosaccharides absorbable by the body in mammals (Seetaloo et al., 2019). The brush-border enzyme complex consists of maltase-glucoamylase, sucrase-isomaltase, lactase, and trehalase (Van Beers et al., 1995). α-Glucosidase activity plays a direct role in the postprandial glucose rise; hence, inhibition of these enzymes is an established approach to glycemic regulation in people with diabetes (Kato et al., 2012; Ahmad et al., 2017). On the other hand, a complete deficiency of lysosomal α-glucosidase causes Pompe disease, highlighting the enzyme’s systemic relevance (Hers, 1963).

## 6 Human α-glucosidases: structure, function, and clinical importance

### 6.1 Intestinal α-glucosidases

Sucrase Isomaltase (SI) is an intestinal brush border enzyme that is an integral part of α-glucosidase and is involved in the final step of carbohydrate digestion by breaking down disaccharides and oligosaccharides into monosaccharides that can be absorbed (Gericke et al., 2017). This enzyme consists of two homologous functional units, sucrase and isomaltase, each a member of the GH31 family and differing in substrate specificity. The common feature of all GH31 enzymes is a consensus sequence, which serves as the catalytic nucleophile (Gericke et al., 2017). Sucrase-isomaltase (SI) and maltase-glucoamylase (MGAM), the two dominant intestinal α-glucosidases, are responsible for cleaving α-1,4 glycosidic linkages that form the backbone of dietary starch (Tannous et al., 2023). Deficiency or a total lack of SI enzyme activity can impair carbohydrate absorption, leading to gastrointestinal symptoms such as osmotic diarrhea, bloating, flatulence, and vomiting (Cohen, 2016). This deficiency is usually caused by changes in the majority of the coding region of the SI gene, which causes congenital sucrase-isomaltase deficiency (CSID) (Alfalah et al., 2009). Human SI is abundant in the intestinal lumen and has broad substrate specificity, hydrolyzing α-1,2, α-1,6, and α-1,4 glycosidic bonds. It accounts for nearly all sucrase activity and approximately 60–80% of maltase activity (Treem, 2012). CSID, also known as genetic sucrase-isomaltase deficiency (GSID) or sucrose intolerance, is a genetic disorder characterized by the absence or reduced activity of the sucrase and isomaltase enzymes, leading to digestive difficulties (Alfalah et al., 2009). Brush-border maltase-glucosidase (MGAM) catalyzes the final breakdown of dietary starch into glucose in the small intestine. It is anchored to intestinal epithelial cells and consists of two homologous GH31 catalytic subunits, the membrane-proximal N-terminal (NtMGAM) and the luminal C-terminal (CtMGAM) (Sim et al., 2008). The domains are anchored to the brush border membrane of the small intestine through an O-glycosylated stalk that extends to the N-terminal domain (Sim et al., 2010). Recombinant enzyme analysis has demonstrated that the NtMGAM exhibits the highest activity specifically against maltose. In contrast, the CtMGAM shows a broader substrate range, including activity against glucose oligomers. The enhanced catalytic efficiency of the C-terminal subunit is attributed to its greater affinity for glucan substrate and the presence of multiple binding configurations within its active sites (Quezada-Calvillo et al., 2008). The MGAM and SI genes evolved through the duplication and divergence of an ancestral gene, and their corresponding N-terminal and C-terminal domains share high sequence identity (60%), whereas N and C-terminal domains within the same enzyme are less similar (Nichols et al., 2003; Sim et al., 2010). Acarbose is the most used α-glucosidase inhibitor, effectively blocking the C-terminal domains of MGAM and SI but less effective against the N-terminal domains (Quezada-Calvillo et al., 2008).

### 6.2 Lysosomal acid α-glucosidase (GAA)

Lysosomal acid α-glucosidase (GAA) or acid maltase is an essential lysosomal hydrolase that degrades glycogen exo-1,4 and exo-1,6-α-glucosidase activity to release glucose. Its cDNA encodes a 952-amino-acid polypeptide with an estimated molecular mass of ∼150 kDa (Hoefsloot et al., 1988). The nascent GAA precursor contains an N-terminal signal peptide that mediates co-translational translocation into the endoplasmic reticulum, where it undergoes N-glycosylation at 7 sites, generating a ∼110 kDa glycoprotein intermediate (Moreland et al., 2005). It is synthesized as a glycoprotein precursor that is delivered to the lysosome via mannose-6-phosphate receptor trafficking and proteolytically processed into its mature, multi-peptide active form (Roig-Zamboni et al., 2017). Pompe disease is a rare, inherited metabolic disorder that is caused by a mutation in the gene encoding GAA, a lysosomal enzyme (Lombard et al., 2014). Loss of functional GAA causes lysosomal glycogen accumulation and subsequent tissue damage, predominantly affecting cardiac and skeletal muscle (van der Ploeg and Reuser, 2008; Raben et al., 2002).

## 7 *Candida albicans* α-glucosidase: a virulence-associated enzyme

### 7.1 Cell wall architecture and glycosylation

*C. albicans* is an opportunistic fungus that typically causes mucosal infections in humans but can disseminate and produce life-threatening systemic disease in individuals with compromised immunity (Mora-Montes et al., 2007). The fungal cell wall serves as the principal interface between *C. albicans* and the host, making it central to host-pathogen interactions. It features an inner scaffold of chitin and β-1,3 and β-1,6-glucans, overlaid by an outer mannoprotein-rich layer, which accounts for approximately 40% of the cell wall in the yeast form (Klis et al., 2001). Mannoproteins, which contain both O- and N-linked oligosaccharides, are the most prominent factors of fungal adhesion, antigenicity, host immune modulation and immune recognition (Bates et al., 2006; Mora-Montes et al., 2007). Fungal O-linked glycosylation involves the attachment of linear α-1,2-mannose oligosaccharides to serine or threonine residues, a modification that is critical for cell wall assembly and pathogenicity. In *C. albicans*, α-glucosidase plays a key role in the biosynthesis and remodeling of cell wall components. When experimental structural data are unavailable, homology modeling serves as an established method for generating three-dimensional protein models based on evolutionary relatedness (Rashid et al., 2023). In the endoplasmic reticulum, the *cwh41* and *rot2* genes encode α-glucosidases I and II, respectively (Mora-Montes et al., 2007). The deletion of *cwh41* or *rot2* has been shown to profoundly affect *C. albicans* growth, leading to the delayed filamentation, shortening of germ tubes, depletion of cell wall mannose content and diminished virulence, and therefore the deletion of α-glucosidase demonstrated the importance of this protein in growth and pathogenicity (Mora-Montes et al., 2007). Several studies on *C. albicans* have revealed that protein glycosylation plays a vital role in fungal virulence and in recognition by the host immune system. Pathogenicity depends on mannosyltransferases involved in the N- and O-linked glycosylation (Buurman et al., 1998; Bates et al., 2006). In addition, golgi-resident proteins that supply GDP- Mannose to be glycosylated, which are encoded by CaCRG4 and CaSRB1, are essential to fungal survival and hence the primary role of glycosylation to fungal cell survival (Herrero et al., 2002).

### 7.2 Role in adhesion, biofilm formation, and immune evasion

Over the last few decades, careful research on the virulence of *C. albicans* has presented a variety of molecular targets and signaling pathways that can be used to establish new anti-fungal and anti-virulence interventions (David et al., 2024). α-Glucosidases may also contribute to biofilm formation and remodeling in *C. albicans* by mediating extracellular carbohydrate processing. Biofilm development in *C. albicans* is characterized by the production of a complex extracellular matrix composed of polysaccharides, proteins, lipids, and extracellular DNA, where carbohydrate-modifying enzymes play a critical role in matrix assembly and structural integrity. Glycosidases, including α-glucosidases, are implicated in the hydrolysis and remodeling of glucan-rich components, thereby influencing biofilm architecture, maturation, and dispersal dynamics. These processes are of particular clinical importance, as biofilm-associated cells exhibit enhanced tolerance to antifungal agents and contribute to persistent infections, especially in hospital environments involving indwelling medical devices. Emerging studies on fungal glycosidases further support their role in modulating host-pathogen interactions and biofilm physiology, highlighting them as potential targets for anti-virulence strategies (Ortiz-Ramírez et al., 2023). The initial important event in *C. albicans* pathogenesis is host epithelial adhesion, which aids colonization and invasion. The increased attachment of hyphal forms is due to the high expression of adhesion proteins, which play a vital role in establishing robust host-pathogen interactions with mannoproteins, the heavily glycosylated substances of the *C. albicans* cell wall (Zhao et al., 2022; David et al., 2024). α-Glucosidase is essential for processing mannoproteins, the heavily glycosylated components of the outer cell wall of *C. albicans*, which are crucial for cell wall integrity and functions (David et al., 2024). Mannoproteins play a major role in adhesion, antigenicity, host immune regulation and recognition by innate immune cells (Mora-Montes et al., 2007). Blocking α-glucosidase activity inhibits the maturation of mannoproteins, weakening cell wall integrity and reducing adhesion to host tissues (David et al., 2024). One such virulence factor of *C. albicans* is biofilm formation, which has been identified to play a significant role in invasive fungal infections (Nobile and Mitchell, 2006). Even with the antifungal treatment, patients with invasive candidiasis still die upto 40% of the time (Wey et al., 1988). The microbial communities that bind to surfaces and are enclosed in an extracellular matrix that shields them against antifungal treatment are called biofilms and are linked to chronic infection (Finkel and Mitchell, 2014). The most common type of bacterial growth is biofilms, which contribute to clinical infections because they exhibit high antibiotic resistance (Chandra et al., 2001). One of the dominant structures of biofilms is the extracellular matrix, which provides a structural network that shields adherent cells against antifungal agents (Borghi et al., 2016). Initial research proved that the *Candida* biofilm matrix is enhanced in highly dynamic flow conditions, and its degree differs significantly across strains and species (Atriwal et al., 2021). Cell wall-associated genes and adhesins in *C. albicans* are important in cell biofilm formation (Finkel and Mitchell, 2014). The formation of *C. albicans* biofilms follows a defined developmental sequence initiated by adhesion of yeast cells to a surface, followed by germ tube formation, microcolony establishment, and progressive filamentation. The development of biofilms occurs in a reverse order, at first, the cells of yeast are attached, then there is germination, microcolony formation and filamentation (Gulati et al., 2018). The inhibition of α-glucosidase has already been demonstrated to inhibit biofilm formation in *C. albicans*. Research shows that acarbose significantly reduces *C. albicans* biofilm formation across various strains, even at nanomolar concentrations of 90–200 nM (David et al., 2024). *C. albicans* has developed multiple strategies to evade detection and destruction by the host immune system (Oliver et al., 2019). In addition, it exhibits high adaptability and mobility, enabling it to escape immune responses and suppress key antimicrobial defense mechanisms (Jiménez-López and Lorenz, 2013). The function of α-glucosidase in this process is to modify the mannoproteins, which control the host immune response and determine the effectiveness with which the innate immune cells recognize the fungus (David et al., 2024). A second immune-escape strategy of *Candida* involves interactions between surface complement-regulating proteins and the release of proteases that degrade components of the complement cascade (Singh et al., 2020). Hypoxia can be an environmental stress that they can cope with by covering their cell wall to evade immune detection and enhance survival. The lactate emitted by neutrophils under low-oxygen conditions further prolongs this masking (Lopes et al., 2018). In addition, the immune defense avoidance and host tissue penetration enabled by the yeast-to-hypha transition allow them to evade immune defenses (Table 2).

**TABLE 2:** Known/putative functions of *Candida*α-glucosidase in virulence.

S. No	Gene/enzyme name	α -Glucosidase regulation	Influenced pathway/function	Host interaction	Experimental model	Clinical relevance	Evidence/notes	References
1	*cwh41*	+	N-linked glycan trimming → affects N-glycan outer-chain elongation and cell-wall mannoprotein maturation.	•Changes in cell-wall glycan composition •Alter immune recognition and adhesion properties.	•Reduced growth *in vitro* attenuated virulence in mice	•Loss → attenuated virulence in systemic infection models •Relevant to host immune recognition	•Null mutants aggregate •Reduced growth •Altered cell-wall carbohydrate composition •Reduced virulence	(Mora-Montes et al., 2007)
2	*rot2*	+	Trimming of N-linked glycans: glycoprotein maturation	•Altered mannoprotein display affects recognition by innate immune receptors	*rot2* mutants created, and *in vitro* cytokine stimulation assays with human PBMCs/macrophages carried out	•*rot2*→ attenuated virulence •Defects in cell-wall integrity can change host responses •Lower pathogenicity	•Genetic deletion leads to misglycosylation •Altered cell wall •Changes in cytokine induction •Reduced virulence in mice	(Sturtevant et al., 1999)
3	*gca1*	+	Environmental carbohydrate degradation → supports growth on complex polysaccharides	•Surface localisation suggests a potential role in adhesion surfaces; •Expressed during oral candidiasis.	Cloning and expression studies RT-PCR detection of GCA1 expression in a rat oral candidiasis model	•Expressed during mucosal infection (oral) •May help colonization in glucose-limited niches	•GCA1 transcripts detected in rat oral candidiasis Gca1p found in cell-wall extracts.	(Sturtevant et al., 1999)
4	*sun41*	+	•Cell-wall biogenesis, cytokinesis •Contributes to extracellular matrix composition.	•Required for adhesion to host tissue •Robust biofilm formation.	Gene deletion mutants in *C. albicans*: Adhesion and biofilm assays, Cell-wall assays.	Loss of *sun41* lowers adhesion and biofilm—traits tied to infection persistence.	•Functions as a glycosidase •Involved in cytokinesis •Cell-wall biogenesis •Adhesion and biofilm formation	(Hiller et al., 2007)
6	*Mal*	+	Supports growth in niches where glucose is limited	•Supports persistence in host niches where alternative sugars are available.	Transcriptomics/expression profiling under different carbon sources	Metabolic flexibility contributes to fitness and may influence virulence.	•Transcriptomic studies show upregulation of maltose transporter, maltase •α-glucosidase under alternative carbon conditions links to adaptation and fitness.	(Cottier et al., 2017)
7	*mig1/mig2*	+	•Regulation of alternative carbon source utilization •Sugar transporter genes •Carbon metabolic flexibility •Glucose repression	•Affects hyphal/biofilm formation •Tolerance to cell wall stress •Damage to host cells	Mutant strains (*mig1, mig2* single & double), growth in different carbon sources; RNA-seq, phenotypic assays	•Mutants show reduced virulence traits •Potential metabolic adaptation in host niches where glucose is limited	•Regulation of α-glucosidase •Regulation inferred from broader carbon •Metabolism repression	(Lagree et al., 2020)

## 8 Comparative structural and functional analysis: human vs. *Candida* α-glucosidase

### 8.1 Domain architecture and sequence divergence

There are four main structural domains of an α-glucosidase, namely, an N-terminal domain, a catalytic (β/α)_8_-barrel domain and two C-terminal domains (Kashtoh and Baek, 2022). The N-terminal part of GAA in humans is a trefoil Type-P domain followed by a β-sheet module (Roig-Zamboni et al., 2017). Although it is not directly involved in catalysis, the N-terminal domain plays an important auxiliary role in enzyme stability, proper folding, substrate interactions, and receptor interactions (Tan et al., 2010). Human GAA features a catalytic α/β-barrel with two inserts after β3 and β4 that shape the substrate binding groove. The general structure of its domain is comparable to that of GH31 enzymes, including MGAM and SI, with an N-terminal trefoil Type-P domain, a 2D sheet domain, a catalytic barrel, and C-terminal proximal and distal 2D sheet domains (Roig-Zamboni et al., 2017). The GAA structure also includes seven N-linked glycan chains, five intramolecular disulfide linkages, which are stabilizing and three unpaired cysteine residues (Deming et al., 2017). ccDNA-derived primary sequence analysis of lysosomal α-glucosidase identifies seven N-glycosylation motifs, all validated through site-directed mutagenesis, with Asn-882 and Asn-925 positioned within a C-terminal propeptide that undergoes proteolytic removal during enzyme maturation (Hermans et al., 1993). Human GAA comprises five structural domains, along with seven N-linked glycans, five disulfide bonds, three unpaired cysteins and three protease-removed loops. The domains include an N-terminal P-type trefoil (N1), a β-sandwitch (NW), a (β/α)_8_ catalytic barrel, and two C-terminal β-sandwitch domains (Deming et al., 2017). One study estimated that the proteins should possess a standard type II membrane topology, consisting of a 20-amino acid N-terminal transmembrane segment with a short 2-amino acid cytosolic tail (Mora-Montes et al., 2007). *Candida* α-glucosidase is a protein required in the N-glycosylation processing pathway and possesses structural features that facilitate its cellular activity and interactions (David et al., 2024). These enzymes have a complicated domain structure that can consist of a variety of subunits and structures preserved among various glycosidases (Mora-Montes et al., 2007). The structural features of *C. albicans* α-glucosidase have been predicted by homology modeling, as the three-dimensional crystal structure of *C. albicans* α-glucosidase is not available. One study has shown that *C. albicans* α-glucosidase is highly similar to human α-glucosidase in structure and activity, and that it has conserved catalytic residues (Glu and Asp) in the active site (David et al., 2024). The human GAA enzyme also includes flexible surface loops, such as the G116-M112 region, which may be proteolytically cleaved and are believed to help form a second substrate-binding pocket (Roig-Zamboni et al., 2017).

### 8.2 Glycosylation differences

Human α-glucosidase is N-glycosylated, a post-translational modification, in which oligosaccharides are glycosylated on the specific asparagine residues (Hermans et al., 1993; He et al., 2024). N-linked glycans attached to the enzyme can adopt high-mannose, complex, or hybrid structures, and phosphorylation of specific mannose residues generates mannose 6-phosphate signals that mediate lysosomal trafficking through the mannose 6-phosphate receptor pathway. In *C. albicans*, N-linked glycosylation involves the attachment of oligosaccharides to asparagine residues during protein processing in the endoplasmic reticulum (Frade-Pérez et al., 2010). Glycosylation of cell wall proteins is critical for modulating epithelial immune responses and can promote apoptosis, thereby contributing to fungal pathogenicity *in vivo* (Wagener et al., 2012).

### 8.3 Inhibitor binding profiles

**FIGURE 4:**
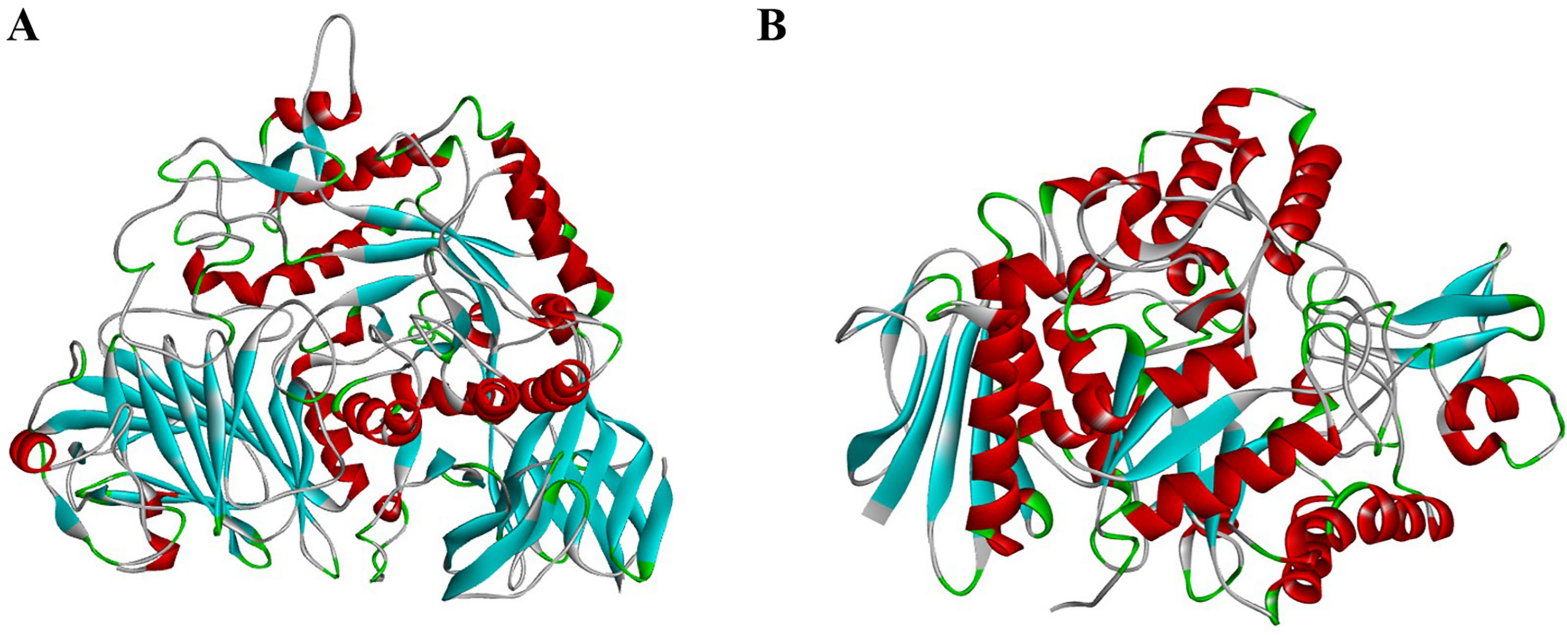
**(A)** Structure of α-glucosidase in human (GH31). **(B)** Structure of α-glucosidase in *C. albicans* (GH13) (Uniprot ID: Q02751) generated by AlphaFold2.

In recombinant human GAA, the primary substrate-binding site is formed by residues located at the C-terminal end of the (β/α)*8*-barrel domain, together with contributions from the N-terminal loop and insertion segments I and II. A secondary pocket in the N-terminal trefoil domain may support processivity, and two distal allosteric sites bind N-acetylcysteine (Roig-Zamboni et al., 2017). The binding of N-acetylcysteine at two distal ends suggests that the enzymes contain allosteric regions (Roig-Zamboni et al., 2017). The carbohydrate-like structures of certain drugs enable them to interact with the carbohydrate-binding site of α-glucosidases from multiple organisms (Dirir et al., 2021). The lack of an experimentally determined three-dimensional structure of *C. albicans* α-glucosidase has limited structure-based inhibitor design; however, homology modeling has been employed to predict its three-dimensional architecture and potential ligand-binding sites (Rashid et al., 2023; Figure 4 and Table 3).

**TABLE 3:** Side-by-side comparison of human vs. *Candida* α-glucosidase.

S. no.	Feature	Human α -glucosidase	*Candida albicans* α -Glucosidase	References
1	Physiological function	•Lysosomal enzyme degrading glycogen to glucose •Encoded by GAA mutations cause Pompe disease	•Processes mannoprotein-linked glycans in the cell wall •Involved in N-glycosylation and cell wall integrity	(GAA Alpha Glucosidase [Homo Sapiens (Human)] - Gene - NCBI, 2026) (David et al., 2024)
2	Active site	•α-Glucosidase mechanism •Active in brush-border or lysosomal contexts	•Conserved catalytic residues (e.g., ASP206, GLU263, ASP338) in binding pocket, similar to human enzyme	(Alpha-Glucosidase Inhibitors, 2016) (David et al., 2024)
3	Structural stability and dynamics	•Human α-glucosidase–inhibitor complex shows stable RMSD ∼0.1–0.25 nm in molecular dynamics simulations	•*C. albicans* complex similarly stable (RMSD ∼0.1–0.3 nm); also compact (Rg, SASA)	(David et al., 2024)
4	Size	110 kDa	66 kDa	(Roig-Zamboni et al., 2017)
5	Amino Acid	•Mature human α-glucosidase (lysosomal) precursor is large •After processing gives several polypeptides (∼110,000 Da precursor)	•*C. albicans* α-glucosidase I used in recombinant assay lacks ∼419 aa at N terminus but retains catalytic activity •Full-length would be larger.	(Moreland et al., 2012) (Frade-Pérez et al., 2010)
6	Inhibition by acarbose	•Acarbose inhibits human α-glucosidase with strong binding	•Acarbose binds strongly to *C. albicans* α-glucosidase with similar interactions and binding affinity	(David et al., 2024)
7	Role in virulence	•Not implicated in virulence •Strictly a lysosomal enzyme for glycogen breakdown.	•Plays a direct role in virulence •Required for N-glycan processing •Cell wall integrity •Inhibition of biofilm formation and adhesion •Hyphal switching	(Mora-Montes et al., 2007)
8	Effect on cell/organism	•In humans, deficiency leads to Pompe disease (glycogen accumulation), •Glycosylation—related trafficking, etc.	•Mutants (cwh41Δ, rot2Δ) in *C. albicans* have reduced growth rates •Altered cell wall composition •Reduced virulence in mice •Altered immune interactions.	(Lim et al., 2014) (Mora-Montes et al., 2007)

### 8.4 Evolutionary divergence and adaptive specialization

α-Glucosidases belong to evolutionarily conserved glycoside hydrolase families, yet their functional roles have diverged significantly across biological lineages in response to distinct metabolic and ecological demands. In higher eukaryotes such as humans, α-glucosidases are tightly regulated enzymes localized to the intestinal brush border, where they facilitate the final steps of carbohydrate digestion and contribute to systemic glucose homeostasis (Sohrabi et al., 2022). In contrast, in *C. albicans*, α-glucosidases are adapted to support metabolic plasticity, enabling the organism to efficiently utilize diverse carbon sources within fluctuating host environments (Lok et al., 2021). This functional divergence reflects evolutionary specialization driven by niche adaptation, in which fungal enzymes are integrated into pathways that support survival, colonization and persistence under host-imposed stresses. Such differences in substrate handling, regulation, and cellular context underscore the potential for selectively targeting fungal α-glucosidases without disrupting host metabolic processes, thereby reinforcing their relevance as anti-virulence targets.

## 9 Phylogenetic and structural superimposition analyses

### 9.1 Evolutionary diversification of α-glucosidase across different biological lineages

The evolutionary diversification of α-glucosidases across different organisms reflects a conserved catalytic framework combined with lineage-specific structural and functional adaptations. In *Saccharomyces cerevisiae*, the presence of multiple glycoside hydrolase genes, such as EXG1, SPR1, and YIR007W, demonstrates that the glucosidase family expanded through gene duplication, followed by functional divergence, producing enzymes with overlapping yet distinct substrate specificities. Although these enzymes retain conserved catalytic residues essential for hydrolysis, variations in substrate preference and catalytic efficiency indicate adaptive specialization after duplication, allowing the organism to utilize a broader range of carbohydrates (Schmidt et al., 2011). A similar evolutionary pattern is observed in thermophilic bacteria such as *Geobacillus*, where α-glucosidase belongs to the GH13 family and shares the conserved (α/β)*8*-barrel fold characteristic of α-amylase–like ancestors. Despite strong conservation of catalytic residues, sequence divergence has resulted in structural modifications that enhance thermostability, reflecting selective pressure to maintain enzymatic activity in high-temperature environments. This demonstrates how evolutionary constraints preserve catalytic function while allowing structural variation to support ecological adaptation (Hung et al., 2005). Further evidence of functional specialization driven by structural divergence is seen in the α-glucosidase from *Halomonas* sp. H11, which also evolved from a GH13 α-amylase–like ancestor, but acquired unique structural features that restrict substrate binding. The presence of an extended β-α loop alters the active-site geometry, conferring strict specificity for maltose, while residues such as Thr203, Phe297, and Gly228 fine-tune substrate recognition without altering the core catalytic mechanism. These findings illustrate that evolutionary modification of peripheral structural elements, rather than catalytic residues, plays a critical role in determining enzyme specificity (Shen et al., 2015). Collectively, these studies support a general evolutionary model in which α-glucosidases originated from a common ancestral glycosidase, followed by gene duplication, structural divergence, and adaptive selection across different organisms. Conservation of the catalytic core maintains glycosidic bond hydrolysis, whereas lineage-specific structural variations enable functional diversification in response to metabolic requirements and environmental pressures. This evolutionary framework provides a basis for understanding the structural differences between human and fungal or bacterial α-glucosidases, supporting the rationale for targeting fungal enzymes selectively in antifungal drug development.

### 9.2 Phylogenetic analysis and structural superimposition of GH13 and GH31 α-glucosidases

**FIGURE 5:**
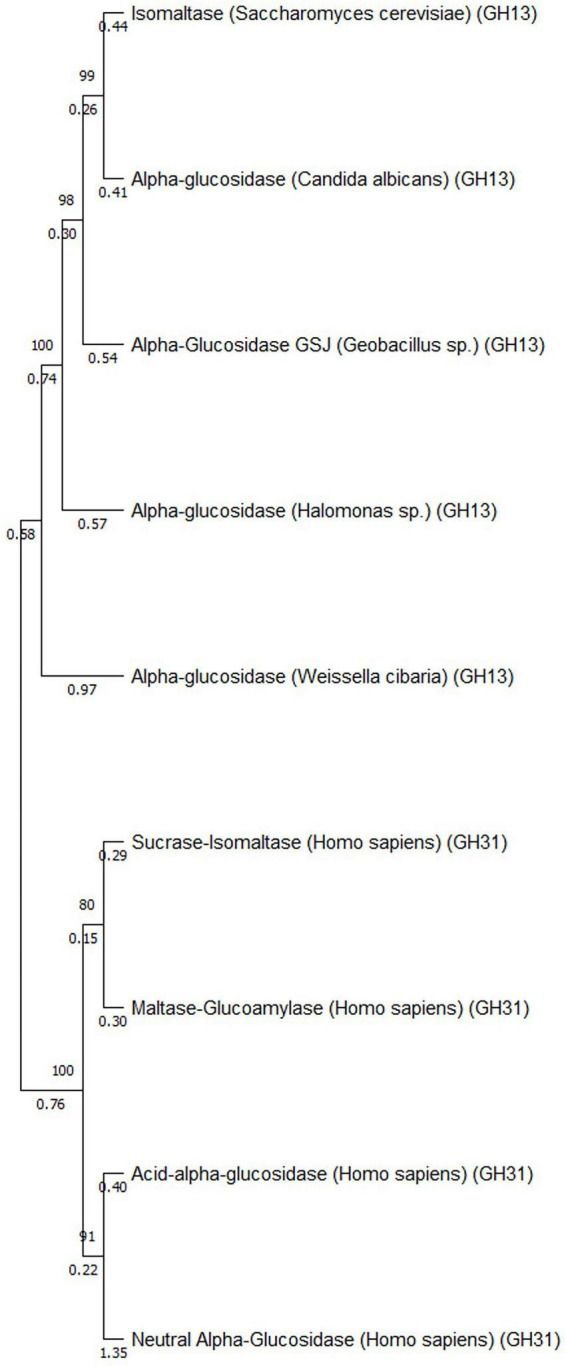
Phylogenetic analysis of different types of α-glucosidase from microbial and human sources.

**FIGURE 6:**
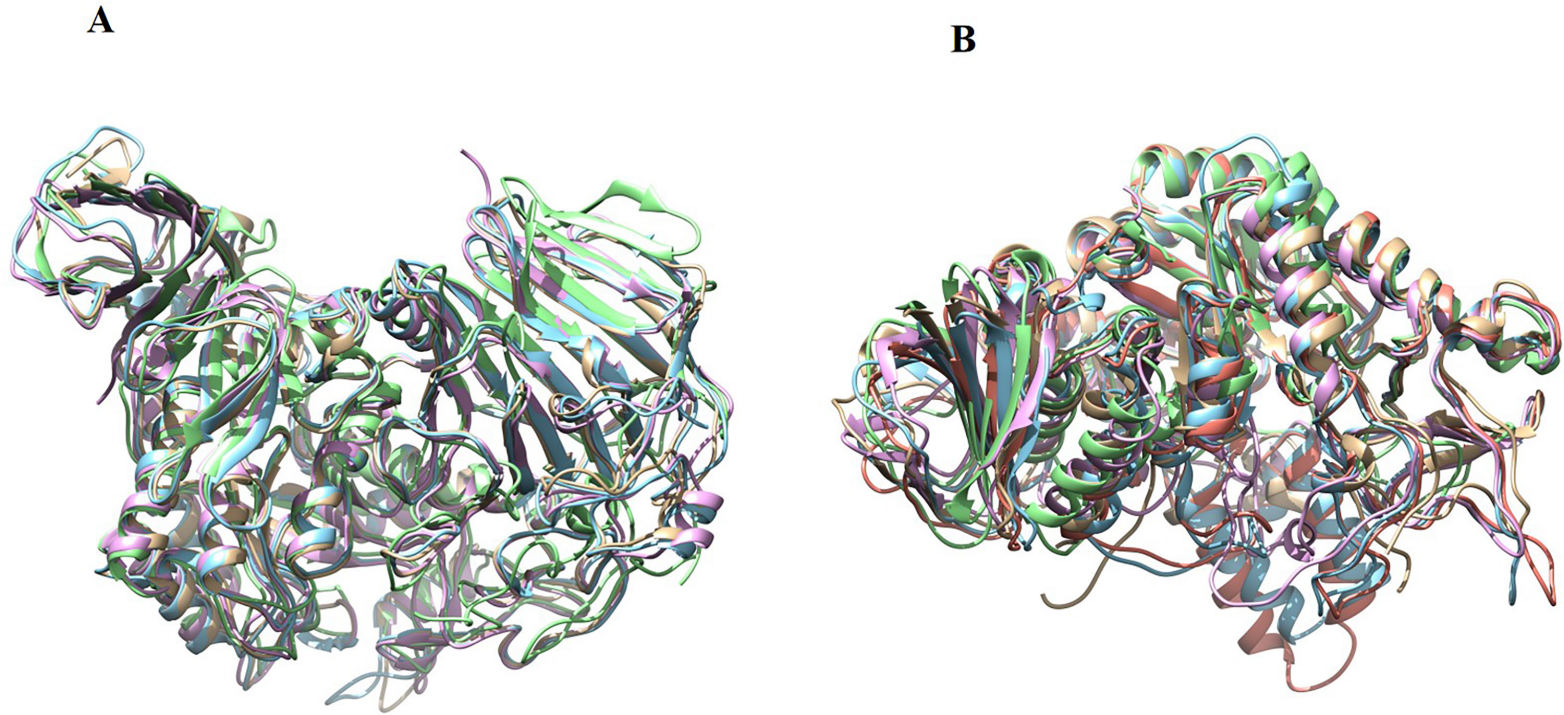
**(A)** Molecular superimposition structure of GH31 including acarbose–maltase glucoamylase (2QMJ), sucrase–isomaltase (3LPO), acarbose-lysosomal acid α-glucosidase (5NN8), and neutral α-glucosidase (8D43), demonstrating conserved structural architecture within the GH31 family **(B)** GH13 family Structural superimposition of GH13 α-glucosidases comprising α-glucosidase GSJ (2ZEO), isomaltase (3A47), α-glucosidase–maltose complex (7DCG), α-glucosidase–maltotriose complex (3WY4), and α-glucosidase from *Candida albicans* highlighting the conserved catalytic fold characteristic of GH13 enzymes and illustrates structural coherence across fungal and bacterial representatives.

The phylogenetic analysis performed using MEGA Software demonstrates clear clustering of α-glucosidase into two evolutionarily distinct families, GH13 and GH31. Fungal α-glucosidase from *C. albicans* groups tightly with bacterial enzymes from *Geobacillus* sp., *Saccharomyces cerevisiae, Weisella cibaria*, *and Halomonas* sp., indicating a strong microbial lineage connection. These GH13 enzymes form a tightly grouped clade, supported by bootstrap values of 80–100, confirming the robustness of their evolutionary relationship. In contrast, human α-glucosidase, including sucrase-isomaltase, acarbose-maltase glucoamylase, acarbose-lysosomal acid α-glucosidase and neutral α-glucosidase, form a clearly separate branch corresponding to the GH31 family. This branch is defined by the unique domain organization and catalytic motifs of GH31 enzymes, which distinguish them from the GH13 family. Together, the strong bootstrap support for separation and structural divergence emphasize significant evolutionary divergence between the GH13 and GH31 families, underscoring the potential for developing selective antifungal inhibitors. To differentiate the structural divergence of GH13 and GH31 family α-glucosidase, we performed schematic structural superimposition analysis within and between the two families using PyMol. Within GH31 Family, which includes sucrase-isomaltase (SI), acarbose-maltase glucoamylase (MGAM), acarbose-lysosomal acid α-glucosidase (GAA) and neutral α-glucosidase (NAG), with the PBD IDs 3LPO, 2QMJ, 5NN8, and 8D43, respectively, are superimposed accurately, indicating a high degree of conservation in their overall fold, domain organization and catalytic framework. Owing to its high-resolution crystallographic quality, we selected MGAM as the reference structure and calculated the Root Mean Square Deviation (RMSD) values and the number of structurally aligned residues (Nres). The analysis showed that SI exhibited the highest structural similarity to the reference, with a low RMSD of 1.105 Å and a high residue overlap of 860 residues, indicating near-identical backbone alignment and strong structural conservation. GAA also aligned well with the reference structure, with an RMSD of 2.178 Å and 837 aligned residues, suggesting overall structural similarity with moderate local deviations. In contrast, NAG showed greater structural divergence, with an RMSD of 4.96 Å and 800 overlapped residues. Since *Candida*α-glucosidase belongs to the GH13 family and no experimentally resolved structure is available, we selected structurally characterized GH13 α-glucosidase from related microorganisms for comparative analysis. These include α-glucosidase GSJ (PDB: 2ZEO; *Geobacillus* sp.), isomaltase (PDB: 3A47; *Saccharomyces serevisiae*), α-glucosidase-maltotriose (PDB: 7DCG; *Weisella cibaria*) and α-glucosidase-maltose (PDB: 3WY4; *Halomonas* sp.), alongside the A lphaFold2 predicted *Candida* α-glucosidase model. The structural superimposition revealed a high degree of intra-family alignment. These proteins superimposed accurately, reflecting conservation of the characteristic (β/α)_8_-barrel catalytic scaffold. Using α-glucosidase-maltotriose as the reference structure, which shows the highest resolution (1.9 Å), RMSD and Nres were calculated to quantify structural correspondence. α-glucosidase GSJ exhibited an RMSD of 5.332Å and 424 aligned residues, reflecting moderate structural correspondence with pronounced backbone deviations. Isomaltase showed an RMSD of 5.823Å and 431 aligned residues, demonstrating even greater divergence from the reference structure. The α-glucosidase-maltose complex displayed an RMSD of 5.654Å and 418 overlapping residues, again supporting a lack of tight structural conservation. The A lphaFold2 *Candida* structure also showed a high RMSD of 5.775Å and 431 aligned residues. However, when GH31 proteins are superimposed with GH13 α-glucosidase, no significant structural alignment is observed, indicating a complete lack of inter-family structural compatibility. This lack of correspondence is quantitatively reflected by an exceptionally high RMSD value of 30.314Å across 564 aligned residues. Although both families retain conserved catalytic residues, such as ASP and GLU, and exhibit similar functions, their overall three-dimensional structures are fundamentally different (Figures 5, 6).

## 10 Therapeutic rationale: targeting *Candida* α-glucosidase

### 10.1 Repurposing α-glucosidase inhibitors

α-Glucosidase inhibitors reduce carbohydrate digestion and absorption by competitively inhibiting α-glucosidase activity (Yin et al., 2014). Several α-glucosidase inhibitors, such as acarbose and voglibose, are effective in regulating postprandial blood glucose levels and are widely used clinically to manage diabetes mellitus (Playford et al., 2013). Several studies show that they can delay or prevent the development of impaired glucose tolerance in diabetes (Bhatia et al., 2019). These inhibitors act on α-glucosidase enzymes, a key factor in the pathogenicity of *C. albicans*, the main organism responsible for causing candidiasis (Chen et al., 2020). The lack of a resolved 3D crystallographic structure for *C. albicans* α-glucosidase has traditionally limited the development of new inhibitors, however, computational modeling is now employed to predict stable 3D conformations (Rashid et al., 2023). Several synthetic inhibitors and natural compounds have been tested for their antifungal activity against *C. albicans*. Nevertheless, despite these studies, considerable limitations have been found with these compounds. Even though miglitol is well established as an inhibitor of α-glucosidase, its use is limited due to gastrointestinal side effects, as well as low efficacy in non-diabetic disorders (Campbell et al., 2000). Likewise, natural compounds have also been tested, though with their own set of challenges. Plant-derived metabolites, such as flavones, exhibit strong antifungal activity against *C. albicans*, inhibiting hyphal growth, biofilm formation, and virulence gene expression. However, their non-specific activity is a major drawback for their use as antifungal agents (Ivanov et al., 2020). Apart from these, even alkaloid-based compounds have been tested, though with limited efficacy. The alkaloids arborinine and graveoline showed antifungal activity against *C. albicans*, but only at relatively high MICs (250–500 μg/mL), suggesting low intrinsic potency. This high dose requirement is a disadvantage for the therapeutic use of the compounds. Thus, optimization strategies may be needed for improved antifungal activity (Kamal et al., 2021). Similarly, terpenoid compounds also show certain limitations in antifungal activity. Terpenoid compounds exhibit antifungal activity against *C. albicans* at high concentrations, suggesting low intrinsic potency. Although terpenoid compounds inhibit hyphal growth, their effectiveness is compromised against mature and mixed-species biofilms. This reduction in antifungal activity is a disadvantage for the compounds. Thus, optimization strategies may be needed for improved antifungal activity (Touil et al., 2020). Thus, there is a certain deficiency in the development of effective antifungal agents. Keeping this in mind, drug repurposing is a promising alternative strategy. Drug repurposing is a strategy for finding new uses for existing drugs, especially for drugs approved by the FDA. This is a faster and cost-effective alternative strategy for drug development. This strategy is gaining attention for the development of antifungal drugs, as it can overcome challenges such as high costs, long timelines, the lack of new chemical entities, and the burden of toxicity testing (David et al., 2024).

#### 10.1.1 Acarbose

Acarbose is a pseudo-sugar and an FDA-approved α-glucosidase inhibitor used to manage type 2 diabetes mellitus. It acts by reversibly inhibiting α-glucosidase enzymes responsible for the breakdown of complex carbohydrates in the intestinal brush border, thereby reducing post-prandial hyperglycemia, lowering the glucose area under the curve, and improving glycosylated hemoglobin levels (Martin and Montgomery, 1996). Acarbose competitively binds to the catalytic site of α-glucosidase due to its structural similarity to natural oligosaccharides, thereby delaying carbohydrate hydrolysis and reducing glucose release in the intestine. This mechanism demonstrates that inhibition of α-glucosidase can effectively modulate glycosidase-dependent metabolic pathways that are conserved across diverse biological systems (Altay, 2022). Consistent with this mechanism, the inhibition of α-glucosidase has been shown to produce significant metabolic effects in clinical settings. Acarbose has been shown to decrease postprandial glucose spikes by 20–30% and reduce insulin requirements by approximately 40% in patients with insulin-dependent diabetes (Dimitriadis et al., 1986). Beyond its glycemic benefits, it is associated with fewer cardiovascular events in high-risk individuals with glucose tolerance and has demonstrated the ability to stabilize carotid plaques (Bell et al., 2008). Importantly, α-glucosidase enzymes are not restricted to human carbohydrate metabolism but are also involved in glycoprotein processing and cell wall organization in pathogenic fungi such as *C. albicans*, suggesting acarbose may also affect fungal virulence-related pathways. Emerging evidence indicates that acarbose can inhibit *C. albicans* biofilm formation, suppress virulence-associated factors, impair yeast-to-hypha transition, and reduce host cell adhesion and invasion even at nanomolar concentrations, likely by disrupting α-glucosidase-mediated N-glycan processing required for proper folding of cell wall glycoproteins and maintenance of cell wall integrity, which are critical for fungal survival and host–pathogen interaction (David et al., 2024).

#### 10.1.2 Miglitol

Miglitol works by reversibly inhibiting α-glucosidase enzymes in the small intestinal brush border, preventing the breakdown of complex carbohydrates, such as oligosaccharides and disaccharides, into absorbable monosaccharides, such as glucose (Hedrington and Davis, 2019). By inhibiting the enzymatic digestion of carbohydrates, miglitol delays glucose absorption and thereby diminishes the postprandial increase in blood glucose levels (Dirir et al., 2021). Unlike its parent compound, acarbose and miglitol are absorbed almost completely in the small intestine. It has little to no impact on fasting blood glucose levels, and its glucose-lowering effect in type 2 diabetes is less potent than that of commonly used sulfonylureas. Long-term studies show that miglitol leads to a modest reduction in HbA1c, typically around 0.3–0.7% from baseline (Sels et al., 1999). Miglitol also reduces body weight, enhances insulin sensitivity and is prescribed to control postprandial hyperglycemia by delaying carbohydrate absorption through α-glucosidase inhibition (Kingma et al., 1992).

#### 10.1.3 Flavonoids

Flavonoids are plant-derived natural compounds known to exert a notable α-glucosidase inhibitory effect, thereby supporting better blood glucose regulation (Fontana Pereira et al., 2011). They are useful in the management of type 2 diabetes by slowing down the digestion of carbohydrates and the uptake of glucose (Barber et al., 2021). These compounds inhibit α-glucosidase through competitive, non-competitive, or uncompetitive mechanisms, depending on whether they bind to the free enzyme or the enzyme–substrate complex (Lam et al., 2024). Various studies have also demonstrated that flavonoids are potent inhibitors of α-glucosidase and moderate inhibitors of α-amylase, making them potentially anti-diabetic with fewer gastrointestinal side effects (Collado-González et al., 2017; Tundis et al., 2016).

#### 10.1.4 Alkaloids

Alkaloids are another significant group of bioactive natural products with strong α-glucosidase inhibitory activity, which is promising as a phototherapeutic agent in the management of type 2 diabetes by reducing carbohydrate hydrolysis and postprandial hyperglycemia (Zafar et al., 2016). A comprehensive survey reported α-glucosidase inhibitory properties in metabolites derived from 53 plant species across 27 botanical families, with approximately 37 structurally characterized alkaloids specifically evaluated for this activity (Dirir et al., 2021; Zafar et al., 2016). The abundance of studies helps emphasize the role of plant-derived alkaloids as new anti-diabetes agents because they have strong enzyme-inhibitory properties (Dirir et al., 2021). It is worth noting that a methanolic extract of the *Commelina communis* was a potent α-glucosidase inhibitor that enabled the isolation of pyrolidine and piperdine alkaloids, including 1-deoxynojirimycin (Kim et al., 1999).

#### 10.1.5 Terpenoids

Terpenoids constitute a major class of bioactive phytochemicals with well-documented α-glucosidase-inhibitory activity and are therefore promising candidates for the management of type 2 diabetes mellitus (Dirir et al., 2021). Due to their structural diversity, terpenoids have significant potential as therapeutic agents, particularly as α-glucosidase inhibitors, and offer an appealing, plant-based alternative to standard antidiabetic agents. Their natural origin also confers a favorable safety profile, which helps promote their application as effective medicines for the treatment of diabetic pathophysiology. A total of 95 terpenoids with α-glucosidase properties have been documented, including sesquiterpenoids, diterpenoids and triterpenoids. In contrast, although synthetic inhibitors like acarbose, mitigol and voglibose are clinically available, their therapeutic utility is often limited by gastrointestinal side effects such as diarrhea and abdominal discomfort. Hence, it emphasizes the need for safer, naturally derived alternatives with improved tolerance (Dirir et al., 2021; Table 4).

**TABLE 4:** Known α-glucosidase inhibitors tested on fungi with IC_50_ values.

S. No	α -Glucosidase inhibitor (g/mol)	Molecular weight (g/mol)	Source/origin	Species of *Candida*	Strains of *Candida*	Mode of inhibition/ mechanism	Reported value (IC_50_/K_i_/effective concentration)	Assay (s)	Clinical/pharmacological relevance	References
1	Acarbose	645.604	FDA-approved synthetic glycomimetic	*Candida albicans*	SC5314, ATCC90028 and BF1	•Targets α-glucosidase of *Candida albicans*, via transcriptomic data. •Interferes with mannoprotein processing in the cell wall •Morphological switching •Adhesion and invasion.	90-200 nM	•Biofilm formation inhibition •Morphological switching •Adhesion/ invasion assays	•Acarbose is already clinically used, so its safety profile is known. •Because of its anti-virulence effects on *Candida*, it could serve as an adjunct therapy for candidiasis. •Reducing virulence rather than killing	(David et al., 2024; David et al., 2024)
2	Liriope muscari extract	855.02	Natural extract (plant).	*Candida albicans*	KCTC 7965	•Inhibits biofilm formation •Blocks yeast-to-hyphae transition •Downregulates hyphal, ECM, and signalling pathway genes	1.56 μg/mL	•Antifungal/anti-biofilm effect alongside α-glucosidase targeting	•Has potential as a natural/herbal anti-biofilm or antivirulence agent •Could be used in combination with antifungals.	(Lee et al., 2024; Lee et al., 2024)
3	Essential oils		Natural plant-derived. Various essential oils	*C. albicans* *C. tropicalis*	–	•Inhibit α-glucosidase and α-amylase *in vitro* •Show antioxidant and antimicrobial activity •*In silico* docking confirms binding of major components to these enzymes.	–	•α-glucosidase inhibition and fungicidal effect •Often reported as dose-dependent activity.	•Potential as natural sources of α-glucosidase inhibitors. •Could be used in coatings, topical antifungals, or in formulations to reduce *Candida* biofilms.	(Jeddi et al., 2023; Jeddi et al., 2023)
4	1-Deoxynojirimycin (DNJ)	163.17	Natural (plants like mulberry, dayflower) and microbial origin (iminosugar).	*C. albicans*	–	–	Addition of 5 mM DNJ suppressed fungal growth by > 50% in maltose media	•Growth inhibition assay •Effect of α-glucosidase inhibition on growth.	•DNJ is known in anti-diabetic/glycosidase inhibition •Could serve as an antifungal growth inhibitor when *Candida* depends heavily on certain sugars.	(Bramono et al., 1995; Bramono et al., 1995)
5	Magnoflorine	342.36	Natural alkaloid, plant-derived.	*C. albicans*, *C. tropicalis*, *C. glabrata*	*C. albicans* SC5314 and clinical isolates	•Inhibits fungal α-glucosidase (55.91% at 50 μg/mL) •Disrupts biofilm formation •Synergistic with miconazole	•MIC = 50 μg/mL •α- glucosidase inhibition = 55.91 ± 7.17% at 50 μg/mL	•Disk diffusion assay •MIC •α-glucosidase activity assay •Biofilm inhibition •Cytotoxicity on HaCaT cells	•Potential antifungal agent •Synergistic with azole drug miconazole •Non-toxic to human cells up to 200 μg/mL.	(Chen et al., 2020; Chen et al., 2020)
6	Quercetin	302.24	Plant-derived secondary metabolites	*C. albicans, C. tropicalis, C. glabrata, C. krusei, C. parapsilosis*	Laboratory and clinical isolates	•Inhibits biofilm, adhesion & invasion; •Induces apoptosis in resistant strains (quorum-sensing) •Inhibits yeast α-glucosidase	•MIC (*C. albicans* SC5314): 128 μM (∼38.7 μg mL^–1^) • MFC > 512 μM. •α-glucosidase: reported K_i_ ≈ 6.3 × 10^8^1 M	•MIC by broth microdilution •XTT reduction assay for biofilms •Adhesion/invasion •*In vivo* VVC mouse model	•Reduces biofilm •Sensitizes resistant strains •Protects VVC mice	(Tan et al., 2023; Tan et al., 2023; Zhou et al., 2024; Zhou et al., 2024)
Known α-glucosidase inhibitors tested on Humans with IC50 values										
**S.No**	**α -Glucosidase inhibitor**	**Molecular weight**	**Source/origin**	**Mode of Inhibition or mechanism**		**Reported value (IC_50_/K_i_/effective concentration)**		**What was measured/assay**	**Clinical/Pharmacological relevance**	**References**
1	Betulinic acid	–	Natural compound	•Docking suggested hydrogen bonding •Hydrophobic interactions with α-glucosidase		–		Molecular docking (CDOCKER) against homology model of α-glucosidase	Proposed as a candidate inhibitor based on binding energy and docking interactions	(Riyaphan et al., 2021; Riyaphan et al., 2021)
2	Oleanolic acid	–	Natural compound	Docking predicted binding to Ser295, Glu270, etc. in α-glucosidase model		–		Docking/molecular modeling	A potential α-glucosidase inhibitor candidate	(Hiller et al., 2007; Hiller et al., 2007)
3	Acarbose	MW ≈ 645 Da	Microbial (Actinoplanes sp.) —marketed drug	Competitive/reversible inhibitor of intestinal α-glucosidases (MGAM, SI)—substrate mimic.		•Sucrase IC_50_ = 1.65 μM •Maltase IC_50_ = 13.9 μM •Isomaltase IC_50_ = 39.1 μM		Inhibition of human intestinal sucrase, maltase, and isomaltase measured by sugar breakdown (HPAE-PAD) in Caco-2 CFE.	•Regulate sugar digestion •Postprandial glycaemia without the side effects.	(Barber et al., 2021; Barber et al., 2021)
4	Miglitol	MW ≈ **207.2** Da	Synthetic iminosugar (1-deoxynojirimycin derivative)—marketed drug	•Reversible inhibitor of intestinal α-glucosidases (maltase-glucoamylase/sucrase) •Binds active site (iminosugar mimic of transition state).		–		•Measurement of post-prandial blood glucose •HbA*1*c in clinical studies	•Used in type 2 diabetes as an adjunct to diet/exercise to reduce post-meal glucose excursions •Modestly reduce HbA*1*c (∼0.3–0.7%)	(Sels et al., 1999; Sels et al., 1999)
5	Voglibose	267.28 g/mol	Synthetic valiolamine derivative (AO-128)	•Competitive inhibitor of intestinal α-glucosidases (sucrase, maltase, isomaltase) •Blocks hydrolysis of oligosaccharides		–		•Inhibition of human intestinal α-glucosidase •Assays on brush-border enzyme activity. •Clinical study also measured endogenous GLP-1 secretion after voglibose.	•Used as an oral antidiabetic drug to control postprandial hyperglycemia •Shown to mobilize endogenous GLP-1 in humans.	(Göke et al., 1995; Göke et al., 1995)

## 11 *In silico* insights

**FIGURE 7:**
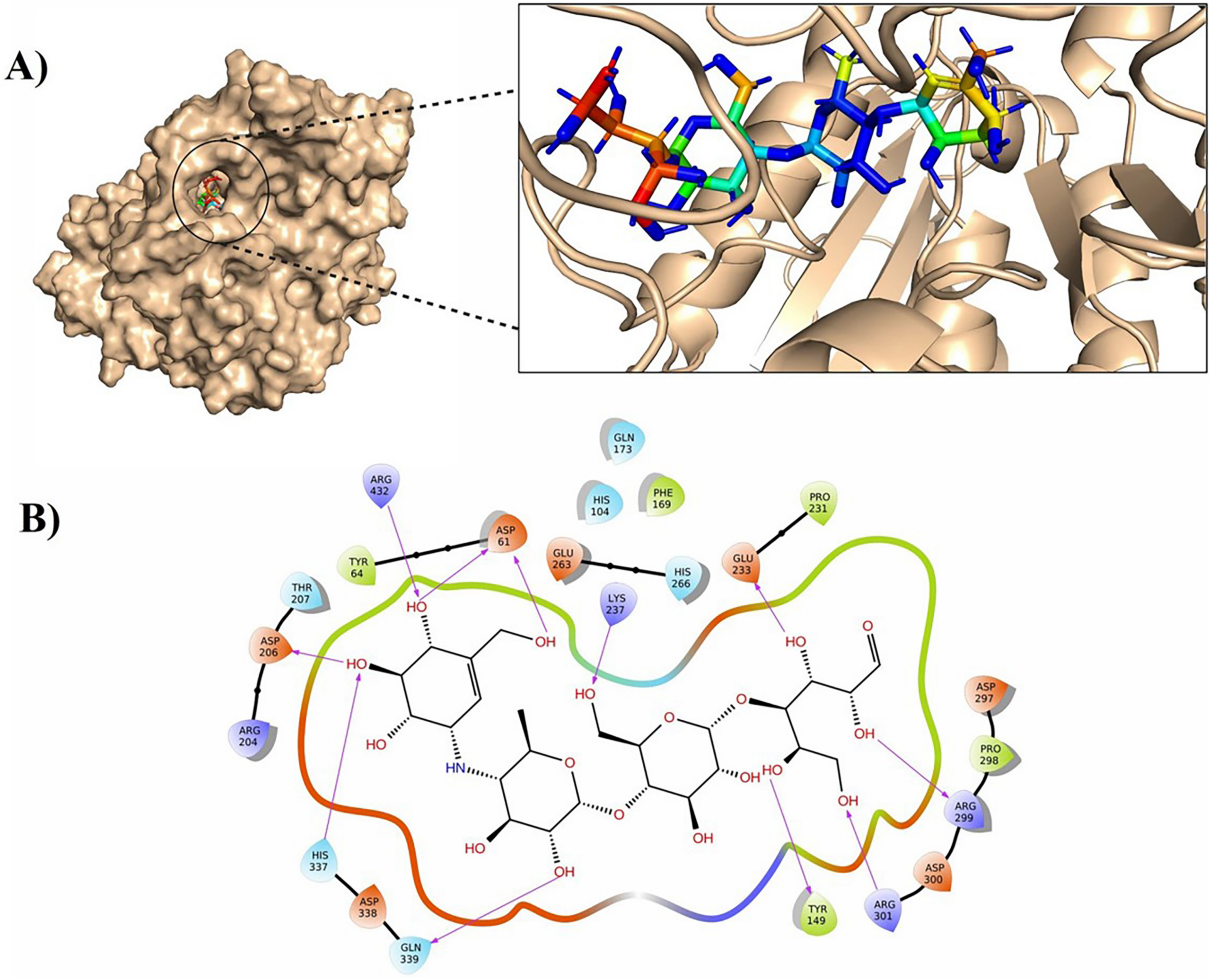
Representation of the docking of the docked acarbose-*C. albicans* α-glucosidase complex interacting with active site residue. **(A)** The three-dimensional docking configuration illustrates the protein displayed in a white-colored surface rendering (left) and a cartoon representation (right), with acarbose visualized as a rainbow-colored stick model within the active site. **(B)** The two-dimensional interaction profile highlights key molecular associations, including hydrogen bond (Purple arrow) with ASP206 and an electrostatic interaction (orange) with GLU263 and ASP338 (David et al., 2024).

*C. albicans* α-glucosidase is a key virulence factor in candidiasis, especially in immunocompromised individuals. The absence of crystallographic or experimentally validated structural data has posed a significant barrier to rational inhibitor development. However, recent *in silico* approaches such as homology modeling and molecular docking have now enabled the efficient prediction, screening and characterization of potential inhibitors from both natural and synthetic compounds (Peytam et al., 2021). Homology modeling is a computational approach used to predict a protein’s 3D structure when no experimental models are available. For *C. albicans* α-glucosidase, this method has enabled the construction of stable 3D conformers through *in silico* analysis (Rashid et al., 2023). The resulting models are evaluated using Ramachandran plot verification and further refined through the molecular dynamics simulation to ensure structural stability. Such a validated model provides a functional framework for rational drug design and the discovery of novel antifungal therapeutics (David et al., 2024). *In silico* docking studies provide valuable insights into the interactions between drug molecules and *Candida* α-glucosidase. This study is useful in forecasting the binding orientations, strength of interaction, and general inhibitory capacity of the compounds to formulate therapies against candidiasis (Mai et al., 2024). The *C. albicans* biofilm formation and virulence are also inhibited by acarbose, a pseudosugar commonly used to treat persons living with diabetes at nanomolar concentrations. It has been shown through docking studies that it can stabilize a complex with α-glucosidase, through interactions with a conserved active site residue (David et al., 2024; Figure 7).

## 12 Translational perspective: from bench to bedside

The study of *Candida* α-glucosidase and its inhibitors has great potential to develop new antifungal medications (Rashid et al., 2023). Future studies will need to address current challenges while utilizing more advanced technologies to accelerate the discovery of antifungal drugs. Another significant drawback of structure-based inhibitor design is that the three-dimensional structure of the *Candida* α-glucosidase active site has not been experimentally determined, and, as such, no real insight into the active-site architecture or the dynamics of inhibitor binding can be obtained (Rashid et al., 2023). Homology modeling methods and *in silico* methods, which are confirmed by molecular dynamics simulations, are useful initial studies on interactions, but they must be supported experimentally (David et al., 2024). Importantly, recent studies using acarbose, including our study, have demonstrated its translational potential against *C. albicans*. One of the best glycomimetic targets identified during virtual screening for fungal α-glucosidase is acarbose, an FDA-approved α-glucosidase inhibitor used to treat type II diabetes. It suppresses biofilm formation by *C. albicans*, reduces virulence factors, interferes with hyphal morphological transition, and disrupts adhesion and invasion of host cells at nanomolar concentrations, demonstrating its therapeutic value (David et al., 2024). Mechanistically, the effect of acarbose on *C. albicans* is justified by transcriptomic findings indicating that there is a high degree of perturbation of the gene expression in relation to virulence, biofilm regulation, and cell wall processes. These findings highlight the role of α-glucosidase in fungal pathogenicity and provide a strong basis for developing new antifungal strategies. However, predictions of computation should be confirmed by experimental tests. *In vitro* α-glucosidase inhibitory assays enable controlled assessment of enzyme inhibition, drug-drug interactions, and methodological consistency (Jia and Liu, 2007). To study the efficacy of inhibitors under physiological conditions, such as on blood glucose levels and systemic safety, and to estimate biologically meaningful IC_50_ values, *in vivo* validation in animal models is required (Wongchum et al., 2023). Computational methods and AI also help increase the rate of novel inhibitor discovery and prioritization by predicting high-affinity molecules and optimizing chemical scaffolds (Abchir et al., 2024).

The increasing prevalence of antifungal resistance and the complexity of fungal infections underscore the urgency of combination therapies. The conventional antifungals are usually constrained by host toxicity, fungistatic response and the development of resistance phenotypes (Spitzer et al., 2017). Combination strategies may also take advantage of synergistic interactions by attacking different biochemical pathways, different molecular sites, or enhancing the accessibility of drugs within the cell, which expands the antifungal activity and may reduce the possibility of resistance developing (Fohrer et al., 2006; Branda et al., 2025). In our previous *in vitro* study, a mucoadhesive vaginal gel formulation of acarbose demonstrated that an optimized delivery strategy is crucial for clinical applicability, achieving targeted local efficacy while minimizing systemic exposure and associated metabolic side effects (David et al., 2024). Conversely, the oral or systemic routes can be limited by pharmacokinetic factors and other potential complications, especially in patients with metabolic comorbidities. The rationalization of delivery method is thus central to defining a desirable therapeutic window that maximizes antifungal therapy’s effect while preserving host safety. Collectively, these findings indicate that inhibition of *Candida* α-glucosidase, including through repurposed agents such as acarbose, represents a promising antifungal strategy. Integration of computational modeling, experimental validation, combination therapy, and targeted delivery provides a robust framework for translating α-glucosidase-based interventions from bench to bedside.

## 13 Limitations in the therapeutic targeting of α-glucosidase

Although α-glucosidase has been proposed as a promising antifungal and anti-virulence target in *C. albicans*, the current body of evidence remains limited, and several critical gaps must be addressed before its therapeutic potential can be fully validated. Most studies demonstrating the effects of α-glucosidase inhibition are restricted to *in silico, in vitro*, or transcriptomic analyses, with limited mechanistic validation in physiologically relevant or pathogenic models (David et al., 2024). For instance, acarbose-mediated inhibition has been reported to reduce biofilm formation, adhesion, hyphal development, and virulence-associated gene expression (Moon et al., 2025). However, these findings are largely derived from controlled laboratory conditions and require confirmation in *in vivo* and clinical settings. Mechanistic insights into how disruption of glycan processing impacts fungal survival and pathogenicity remain incomplete, although impaired glycoprotein maturation and compromised cell wall integrity have been proposed. Furthermore, the high structural conservation within glycoside hydrolase families raises concerns regarding target selectivity and potential off-target effects on host enzymes. The metabolic adaptability of *C. albicans* presents an additional challenge, as compensatory pathways may mitigate the effects of single-enzyme inhibition. From a translational perspective, the lack of *in vivo* and clinical studies further limits progress in this area. Collectively, addressing these challenges will require integrated approaches combining structural biology, functional genomics, selective inhibitor design, and infection models to establish α-glucosidase as a viable antifungal target.

## 14 Conclusion and future outlooks

A comparative study of human and *Candida* α-glucosidases reveals structural disparities that can be strategically exploited to develop antifungal drugs. From an evolutionary and functional perspective, these enzymes belong to conserved glycoside hydrolase families but have undergone lineage-specific diversification, resulting in differences in domain organization, global structural alignment, and substrate specificity that may allow selective therapeutic targeting. Despite catalyzing key processes in carbohydrate metabolism, there are significant differences in the three-dimensional structures, active-site geometries, and substrate-binding characteristics of the two enzymes, suggesting that achieving selective inhibition depends on their molecular basis. Structural differences between fungal and human α-glucosidases reduce the likelihood of cross-reactivity with host enzymes, thereby minimizing potential cytotoxic effects and improving the selectivity profile of targeted inhibitors. These observations support the concept that *Candida* α-glucosidase is a potentially druggable antifungal target, although further structural and functional validation is required to confirm its suitability for therapeutic intervention. In the context of the increasing global prevalence of antifungal resistance and the limited number of available antifungal drug classes, identifying novel, selectively targetable fungal enzymes remains a major priority in antifungal research. The idea of targeting *Candida* α-glucosidase represents a relatively new approach that may help to overcome common resistance mechanisms by interfering with essential glycan-processing pathways involved in fungal growth, cell wall integrity, and virulence regulation. However, current evidence linking α-glucosidase inhibition to reduced virulence or therapeutic efficacy in *C. albicans* remains limited, with most findings derived primarily from computational approaches that may not fully reflect *in vivo* complexity. While molecular docking and structural comparisons provide valuable preliminary insights into binding interactions and selectivity, they are inherently limited in accurately predicting enzyme dynamics, cellular context, and functional outcomes.
